# Supplementary material for: Visual salience is affected in participants with schizophrenia during free-viewing
Source: Sci Rep. 2024 Feb 26;14:4606. doi: 10.1038/s41598-024-55359-0 (PMC10897421; doi:10.1038/s41598-024-55359-0)
Supplement: Supplementary file 1 — Supplementary Information. [file 41598_2024_55359_MOESM1_ESM.docx]

SUPPLEMENTAL INFORMATION

SUPPLEMENTAL TEXT

**Consideration of resampling scheme.** Considering the possibility that resampled data could affect the results of the statistical analysis, the resampling procedure was repeated 100 times in the statistical analysis with the full model using age-matched control data (Figure 1C, leftmost). For the interaction term, the range of F values was 5.8*10^-5^ - 1.07 and that of P values was 0.30 - 0.99. For the main effect for the participant group in the model without interaction, the range of F values was 19.0 - 36.9 and that of P values was 8.4*10^-9^ - 2.2*10^-5^. Thus, differences in the choice of resampled data do not affect the result of the statistical analysis. Thus, one of the resampled data was selected and used throughout the analysis in the main text.

**Consideration of random sampling scheme.** In Figure 1D, we also examined whether the mean salience values were higher than the chance level, here defined as the expected value from random sampling, i.e., 1.95 * 10^-4^ (= 1 / (80*64) ). In all cases of the four models and two participant groups, they were significantly higher than chance (p < 10^-12^, Wilcoxon signed-rank test with Bonferroni’s correction for multiple comparisons). This confirms that in all models and all participant groups, the participants’ gazes were directed to the salient locations in the test images.


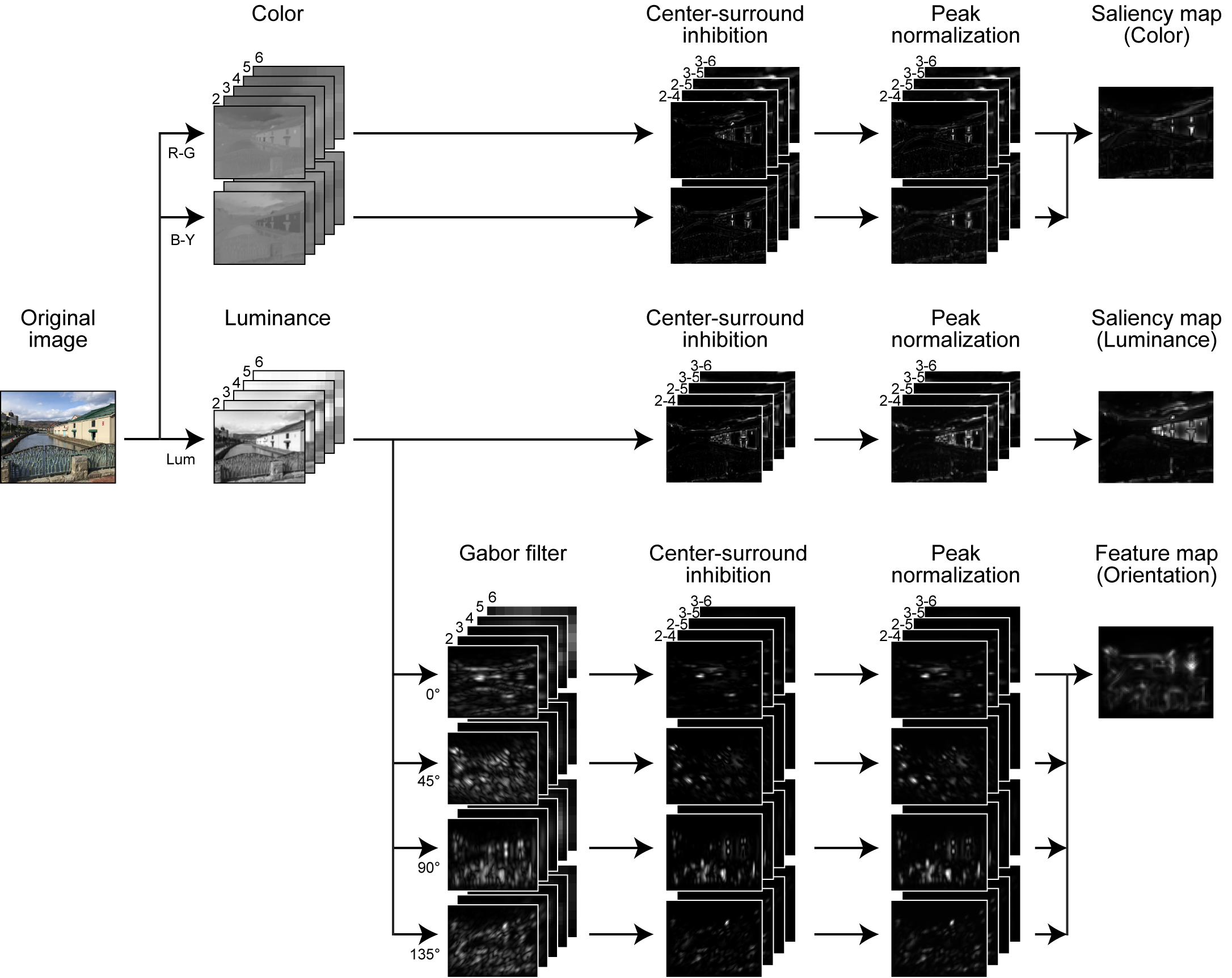


**Figure S1. The computational stages in the Itti-Koch saliency model.**

The Itti-Koch saliency model computes the saliency map of three features, color, luminance, and orientation. The intermediate maps for the orientation feature were used for the analysis in Figure 3. See Methods for details and abbreviations.


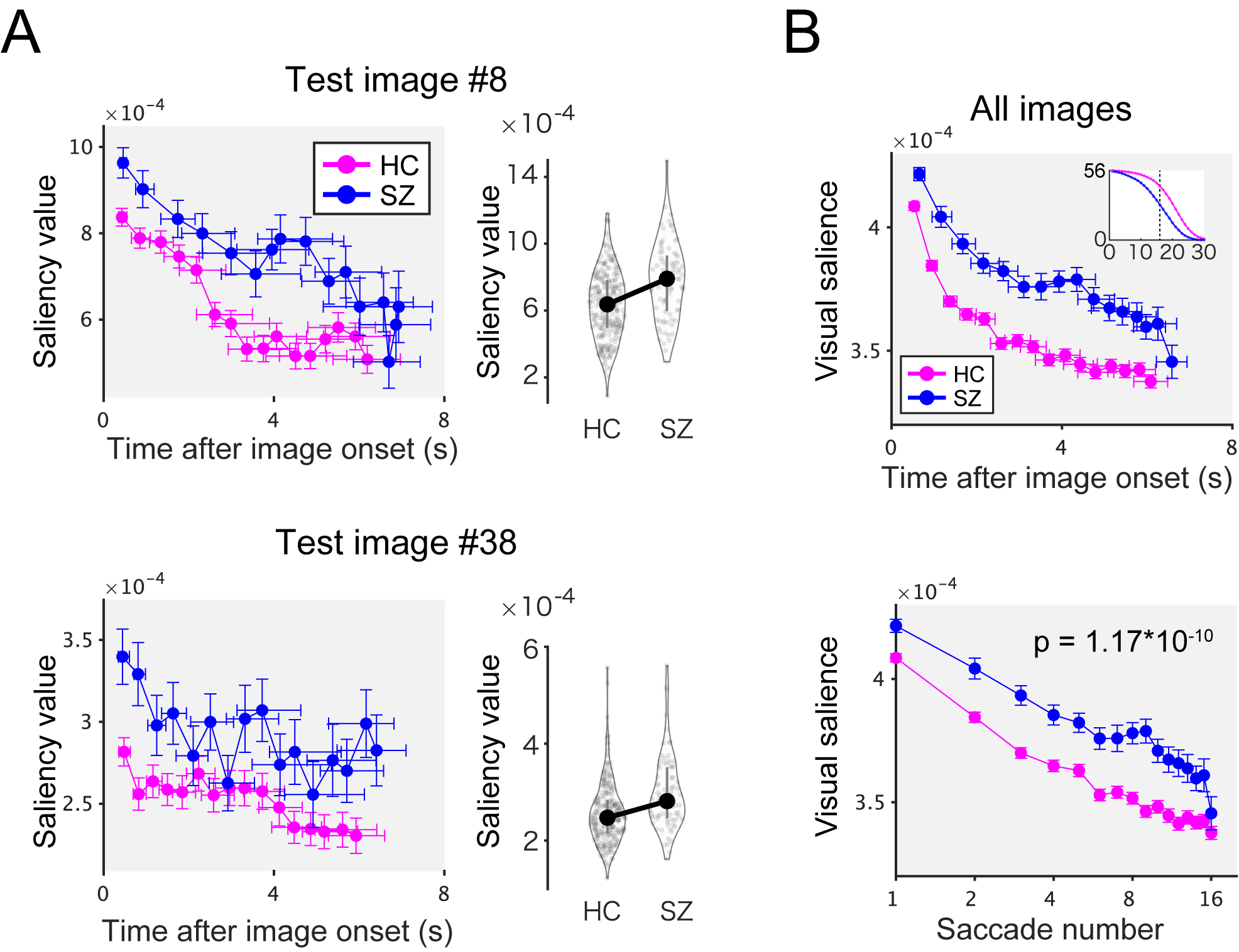


**Figure S2. Salience-guided eye movement are affected single images (A) and across all images (B).**

(A) Left: The saliency values averaged across participants are plotted over time for test images #8 (top) and #38 (bottom). Symbols denote the median. Vertical error bars denote standard errors across participants. Horizontal error bars denote the first and the third quartile. Magenta, HC; blue, SZ. Right: the saliency values averaged across participants and saccades plotted as a violin plot.

(B) Top: The saliency values averaged across test images and participants are plotted over time after image onset. Magenta, the healthy controls (HC; n=252); blue, the participants with schizophrenia (SZ; n = 82). Each point denotes the mean salience value for each saccade (1st through 16th). The symbols and error bars are in the same manner as (A). Inset: number of images obtained for each number of saccades (up to 56) during the 8-sec viewing period. The dotted line denotes the cutoff point (16th saccades). See Methods for details. Bottom: Same as top but the data are plotted across saccade numbers on a log scale. Numbers on the plots denote P values for the main effect of the participant group.


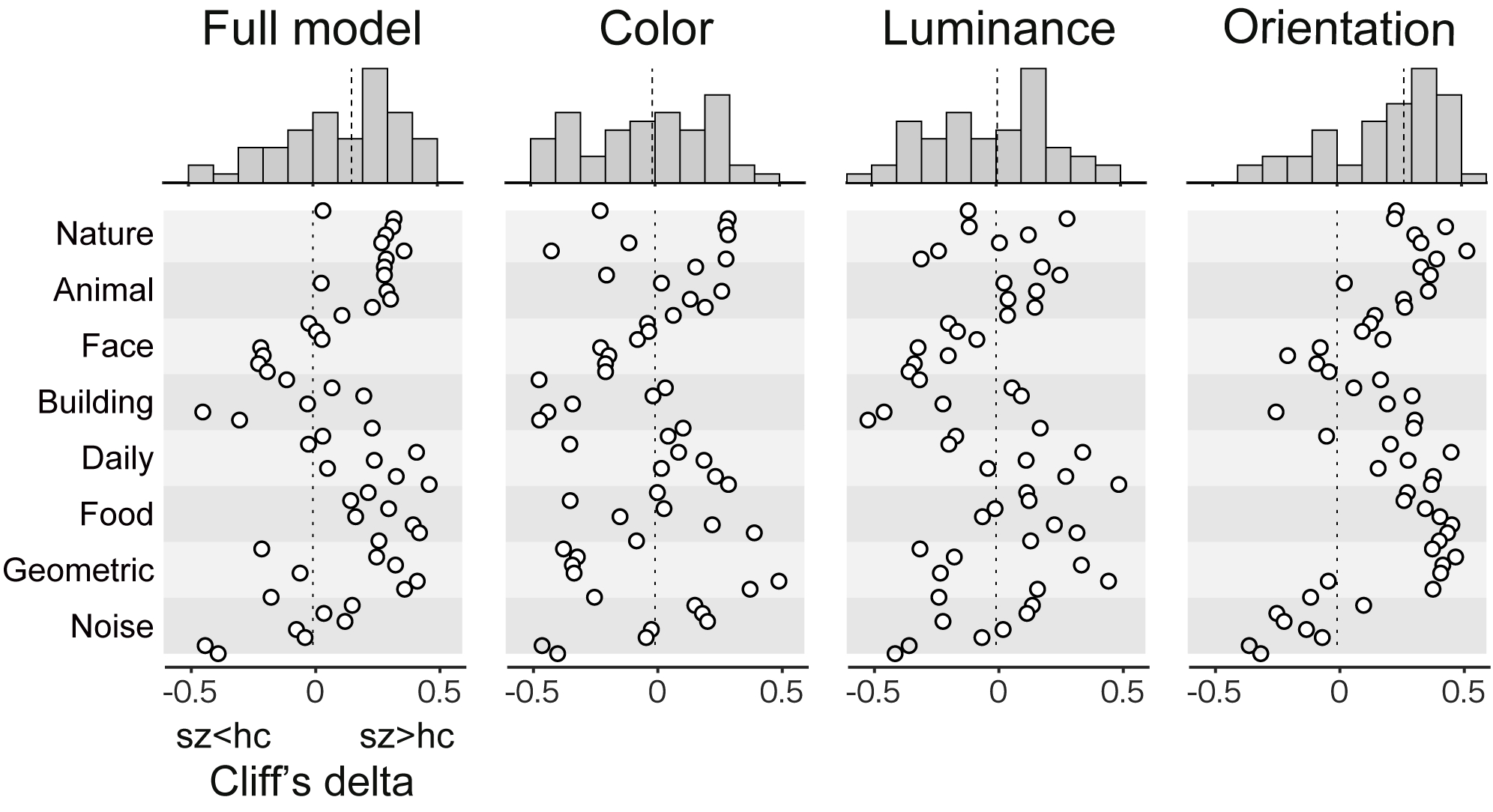


**Figure S3. Image category does not affect the effect of orientation salience.**

Effect sizes (Cliff’s delta) for the test of difference in time-averaged saliency values (Figure 1D) were evaluated for each image. Top: histograms of effect sizes.

**
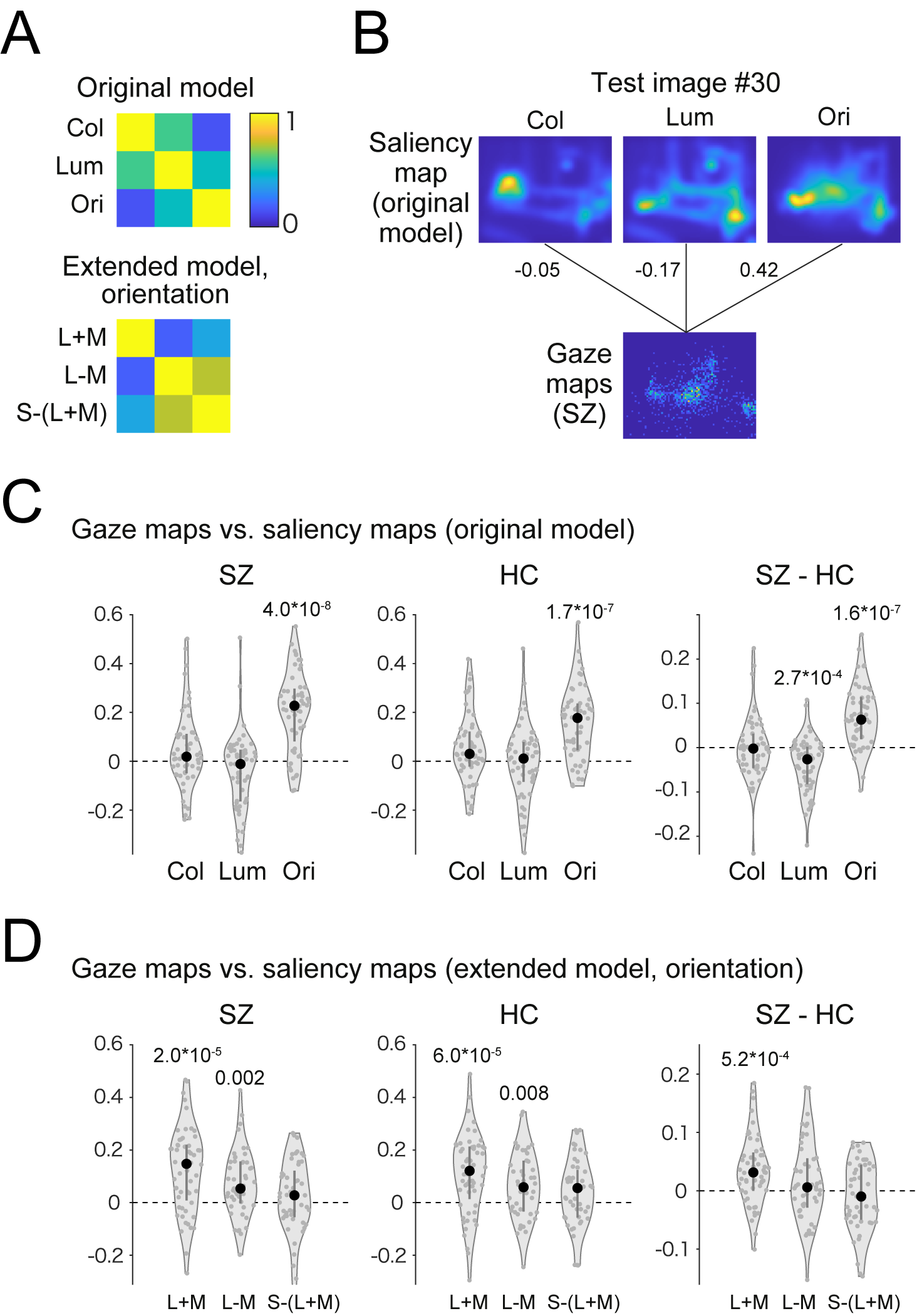
**

**Figure S4. Saliency maps for orientation are correlated with gaze maps.**

(A) (Top) Colors indicate the median of partial correlation coefficients between saliency maps of the original model (top) and the extended six-channel model (bottom).

(B) Examples of saliency maps and gaze maps for test images #30. SZ, the participants with schizophrenia; HC, the healthy controls (age-matched resamples); SZ-HC, the difference between SZ and HC. The numbers indicate partial correlation between the maps. Black for correlation with SZ gaze map, Gray for correlation with HC gaze map.

(C) Violin plots of the partial correlation between the gaze maps and saliency maps (original model). Symbols are the same as in Figure 1D.

(D) Violin plots of the partial correlation between the gaze maps and saliency maps (the extended six-channel). Only the correlations with orientation maps are shown. Numbers in (C) and (D) indicate P values for a significant difference from zero (Wilcoxon signed-rank test after Bonferroni correction). Only the P values less than 0.05 are shown.


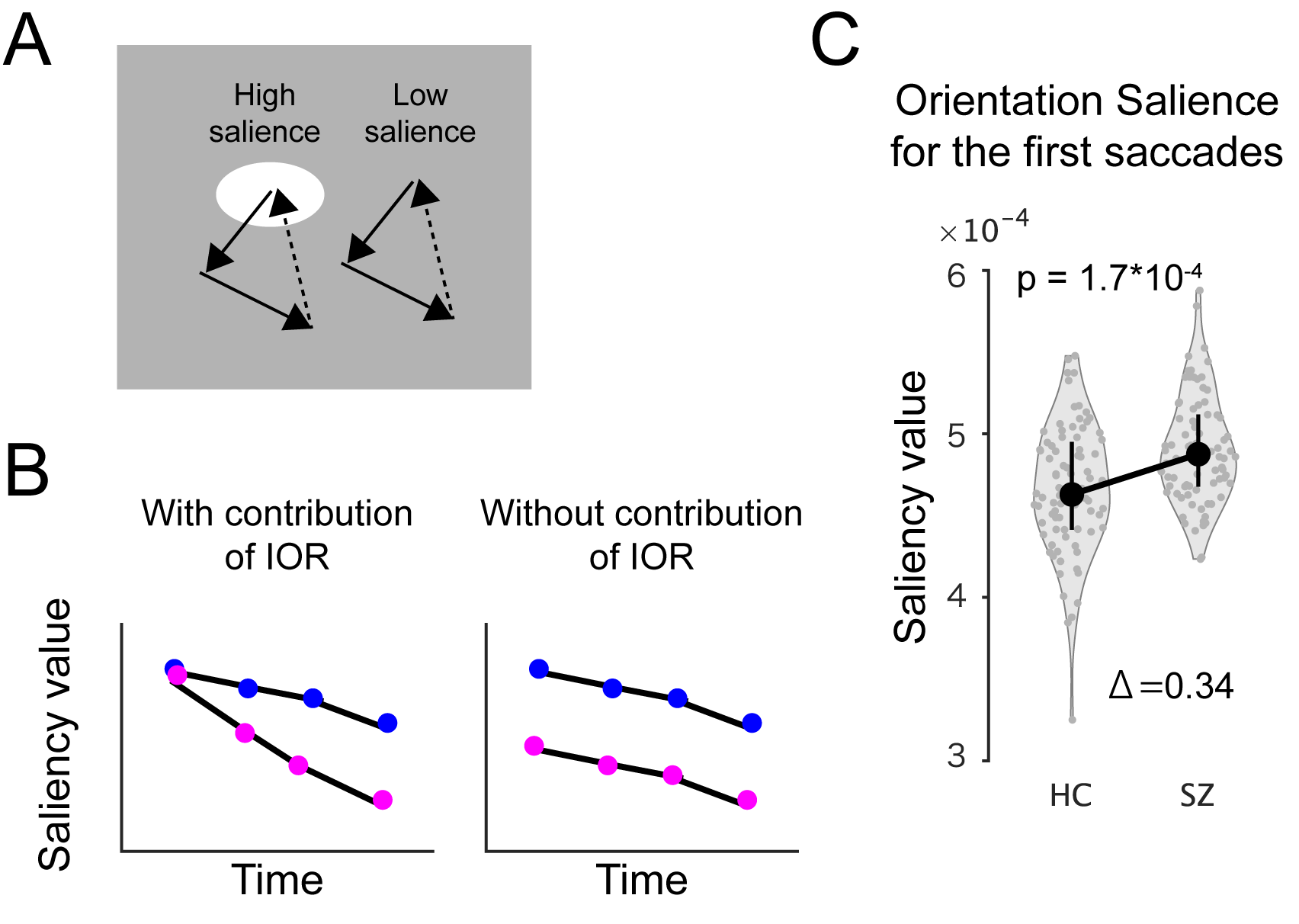


**Figure S5. Inhibition-of-return (IOR) cannot explain the effect of orientation salience.**

(A) Possible scheme for a relationship between the return saccades (dotted line) and the visual salience of the test images (shown in brightness).

(B) Possible time courses of salience values when IOR contributes to salience-guided eye movements (left) and when IOR does not contribute to salience-guided eye movements (right). Our results in Figure 1C support the latter.

(C) Violin plots of the mean orientation salience at the first gaze for each 8-second viewing time. The same notation as Figure 1D.

|  | Subjects with schizophrenia  (n = 82) | | | Age-matched healthy controls  (n = 82) | | | Statistics | |
| --- | --- | --- | --- | --- | --- | --- | --- | --- |
|  | Mean ± SD | | | Mean ± SD | | | P value | Effect  size |
| Sex (male/female) | 42 | / | 40 | 35 | / | 47 | 0.28 | 0.09 |
| Age (years) | 35.1 | ± | 12.4 | 35.2 | ± | 12.3 | 0.94 | -0.01 |
| Education (years) | 13.8 | ± | 2.6 | 15.1 | ± | 2.1 | **5.0×10^-4^** | 0.30 |
| **Symptom-related** | | | | | | | | |
| Onset age (years) | 24.4 | ± | 11.4 | NA |  |  | NA | NA |
| Duration of illness (years) | 10.7 | ± | 8.9 | NA |  |  | NA | NA |
| CPZ equivalent (mg/day) | 600.6 | ± | 545.2 | NA |  |  | NA | NA |
| Cognitive decline | 13.4 | ± | 12.8 | NA |  |  | NA | NA |
| PANSS positive symptoms | 19.1 | ± | 6.1 | NA |  |  | NA | NA |
| PANSS negative symptoms | 20.8 | ± | 6.1 | NA |  |  | NA | NA |
| PANSS general psychopathology | 43.8 | ± | 12.0 | NA |  |  | NA | NA |
| PANSS total | 83.7 | ± | 23.2 | NA |  |  | NA | NA |
| **Cognitive tests** | | | | | | | | |
| WAIS-3 FIQ | 88.0 | ± | 17.9 | 112.6 | ± | 10.2 | **8.1×10^-18^** | 0.81 |
| WAIS-3 VC | 94.3 | ± | 17.2 | 112.5 | ± | 12.9 | **8.5×10^-12^** | 0.71 |
| WAIS-3 PO | 88.1 | ± | 18.0 | 106.1 | ± | 11.6 | **1.2×10^-10^** | 0.67 |
| WAIS-3 WM | 89.6 | ± | 17.2 | 111.1 | ± | 14.1 | **1.4×10^-13^** | 0.76 |
| WAIS-3 PS | 82.3 | ± | 18.3 | 110.1 | ± | 12.4 | **3.7×10^-18^** | 0.87 |
| WMS-R VerM | 89.0 | ± | 21.5 | 114.8 | ± | 13.6 | **1.7×10^-13^** | 0.73 |
| WMS-R ViM | 86.3 | ± | 18.9 | 105.5 | ± | 8.2 | **1.7×10^-12^** | 0.70 |
| WMS-R GM | 86.4 | ± | 21.6 | 114.0 | ± | 12.4 | **2.0×10^-15^** | 0.78 |
| WMS-R AC | 96.3 | ± | 15.0 | 112.0 | ± | 10.9 | **1.5×10^-10^** | 0.64 |
| WMS-R DR | 83.7 | ± | 21.7 | 109.7 | ± | 12.2 | **8.0×10^-14^** | 0.74 |
| UPSA-B social | 68.7 | ± | 16.9 | 83.0 | ± | 10.0 | **1.2×10^-08^** | 0.55 |
| SFS | 101.2 | ± | 31.0 | 148.5 | ± | 19.6 | **3.5×10^-19^** | 0.85 |
| Work hours | 13.0 | ± | 17.2 | 36.2 | ± | 16.9 | **1.4×10^-13^** | 0.71 |
| **Saccade related properties** | | | | | | | | |
| FV MainSeq a | 481.1 | ± | 116.5 | 452.6 | ± | 101.1 | 0.09 | -0.15 |
| FV MainSeq b | 9.8 | ± | 4.9 | 9.7 | ± | 3.1 | 0.55 | 0.05 |
| FV MainSeq c | 28.7 | ± | 9.8 | 28.4 | ± | 7.6 | 0.84 | 0.02 |
| FV Sac number | 16.1 | ± | 5.0 | 21.3 | ± | 4.6 | **9.5×10^-11^** | 0.59 |
| FV Sac amplitude | 2.9 | ± | 1.2 | 3.3 | ± | 0.8 | **0.02** | 0.21 |
| FV Scanpath Length | 72.6 | ± | 29.1 | 109.5 | ± | 22.9 | **5.9×10^-14^** | 0.68 |

**Table S1. Demographic data (Related to STAR methods)**

The “Statistics” column denotes statistical tests for the difference between SZs and healthy controls. All P values are the results of the Wilcoxon rank-sum test for two independent samples, except for the “sex” row, where a χ^2^ test for a 2 x 2 contingency table is performed. P values in bold denote significant differences after correction of multiple comparisons by FDR (P < 0.05). Cliff’s delta is shown as the effect size. Cramer’s V is shown as the effect size for the χ^2^ test. PANSS: Positive and negative syndrome scale. WAIS-3: Wechsler Adult Intelligence Scale 3rd edition; WMS-R: Wechsler Memory Scale-Revised; UPSA-B: UCSD Performance-based Skills Assessment - Brief; SFS: Social Functioning Scale; FV: free-viewing; Sac: saccade. MainSeq a, b, and c: parameters obtained from fitting to the main sequence relationship. See Methods for details.
